# “I Feel It In My Heart When My Parents Fight”: Experiences of 7–9-Year-Old Children of Alcoholics

**DOI:** 10.1007/s10560-018-0544-6

**Published:** 2018-04-20

**Authors:** Agneta Tinnfält, Karin Fröding, Madelene Larsson, Koustuv Dalal

**Affiliations:** Department of Health Sciences, Örebro University, 701 82 Örebro, Sweden

**Keywords:** Children of alcoholics, COA, Qualitative study, Parental alcohol abuse, School children, Vignette

## Abstract

Children are vulnerable when exposed to parental alcohol abuse. Although much is known about children of alcoholics (COA), research examining the experiences of younger COA is scarce. To gain knowledge of the consequences for these children, it is important to ask the children themselves. This study explored the consequences for a child of having an alcoholic parent, from the point of view of 7–9-year-old COA. Eighteen children were interviewed, whose alcoholic parent was undergoing treatment, using a vignette. In the analysis, using qualitative content analysis, the findings show that the children of this young age had much experiences and took a great responsibility for their alcoholic parent, and the family. The most significant feeling of the children was a feeling of sadness. They tried to control the situation in different ways. They wished for a change in the future, but despite problems in the family they described things they did together with a loving parent. Implications include the importance of listening to and supporting all COA, also children as young as 7–9 years old. Further studies should address the support that can and should be offered to COA.

## Background

Decades of research have revealed that children who grow up in families with parental alcohol problems are at risk of several psychological, behavioral, cognitive, and social problems (Brunnberg, Eriksson, & Tinnfält, 2007; Christensen & Bilenberg, 2000; Park & Schepp, 2015). These risks include early onset of alcohol problems and substance abuse, and drinking more alcohol than other young people (Chalder, Elgar, & Bennett, 2005; Chassin, Pitts, DeLucia, & Todd, 1999; Hussong et al., 2008; Wong et al., 2006). Research also indicates that children of alcoholics (COA) are more likely to themselves become alcoholics in later life, and inter-generational transmission of alcohol addiction is well documented among COA (Johnson & Leff, 1999). The literature indicates that COA are exposed to biological (genetic) as well as environmental (caregiver-related) risk factors for several social, familial, and behavioral problems (Edwards, Eiden, & Leonard, 2006; Park & Schepp, 2015).

Parental alcohol problems affect significant numbers of children; up to 30% of young people grow up with at least one parent with a drinking problem (Cuijpers, 2005; Laslett, Ferris, Dietze, & Room, 2012; Manning, Best, Faulkner, & Titherington, 2009). In Sweden, approximately one-fifth of children have parents with alcohol problems (Elgan & Leifman, 2013; Ljungdahl, 2008). Worldwide, up to 40% of children are affected by domestic violence (Evans, Davies, & DiLillo, 2008; Kassis, Artz, Scambor, Scambor, & Moldenhauer, 2013; Zhu & Dalal, 2010), including violence where parental alcohol problems are the cause.

Studies among COA have focused on toddlers, preschoolers and adolescents, but less on children in middle to late childhood. COA display lower self-control during their toddler and pre-school phases (Eiden, Edwards, & Leonard, 2007), and a multitude of studies investigating the adolescent children of problem-drinking parents report increasing problems when COA reach their adolescence (Chassin et al., 1999; Hussong et al., 2008; Park & Schepp, 2015). However, information about children in middle to late childhood is less abundant.

The developmental period from 7 to 9 years is crucial for child development in terms of both self-regulation and external behavior (Park & Schepp, 2015). Middle childhood represents the starting point for logical thinking. Feelings of shame and guilt are known to occur as part of developing a conscience especially during this period of life. Middle childhood is a period when children need to understand themselves and their surroundings (Schofield, 2006). The child starts to think about the future and also becomes more independent from parents. In middle childhood the child can more easily describe experiences and become more concerned about others (Centers for Disease Control and Prevention, 2017). This period of childhood thus warrants further study, including eliciting the children’s own perspectives on the circumstances under which they live.

Family and parents are very important to all children and adolescents. According to the ecological system suggested by Bronfenbrenner (1979, 2000), the dyad between the parents and the child is most important for the development of the child. If any part in the whole system is changed, this will affect the child. The UN Convention on the Rights of the Child (United Nations, 1989) states that the family is the most natural environment for the child, and this is acknowledged in public health research (Blair, Stewart-Brown, Waterston, & Crowther, 2010). Adolescents, when asked, declare that parents and family are very important (Johansson, Brunnberg, & Eriksson, 2007; Tinnfält, Jensen, & Eriksson, 2015). A great fear of adolescent COA is that they will not be allowed to continue living with their parents if the family situation is not good (Barnard & Barlow, 2003; Kroll, 2004; Tinnfält, Eriksson, & Brunnberg, 2011). Parents are important to their children, irrespective of being alcoholics or non-alcoholics.

There seems to be a lack of studies examining how children and adolescents themselves describe the experience of being a COA. Hill (2015) argues that little is known about the lives of COA from their own point of view. She studied 30 children and young people aged 9–20 qualitatively, using several methods. The results revealed that although the COA knew a lot about their parents’ alcohol problems, they made their own decision about what to reveal. It seemed easier to talk about a third person, distancing the situation from their parents. These children and adolescents were mostly concerned about the alcoholic parent, and not about the impact on the family or themselves (Hill, 2015). In another study children aged 7–13 were interviewed after participating in a program to support COA, the issue of resilience was analyzed. The authors reported that the children were clear that adults should not drink, and that tangible assets were not important (Moe, Johnson, & Wade, 2007). Adolescent COA are reluctant to disclose their parents’ alcoholism to adult outsiders, unless these adults ask them explicitly about their home situation and listen carefully to their answers (Tinnfält et al., 2011). Adolescent COA described what it is like living with an alcoholic parent. The adolescents questioned their own worth, as a result of shame and guilt. They felt as though they were impossible to love, and that they were not accepted for the person they were (Murray, 1998). In a study from England, Templeton, Velleman, Hardy, and Boon (2009) interviewed eight young people aged 12–18 from five families about their experiences; the results showed that their lives were uncertain and inconsistent, and that verbal and sometimes physical aggression occurred in the families (Templeton et al., 2009). Female adolescents revealed that their role in the family changed from being a child to taking the parental role, a process called parentification (Hedges, 2012). We need more information on how children, especially younger children, describe their situation being COA, and the consequences that they experience.

It has been known for many years that COA are at risk of several problems. There is an increasing amount of research on adolescent COA’s descriptions of their lives, but this does not seem to be the case for younger children. To create better understanding and to improve the support for younger children, we need to listen to children and explore their point of view. The aim of this study was to explore the consequences for a child of having an alcoholic parent, from the point of view of 7–9-year-old COA.

## Methods

This qualitative study used individual interviews, and qualitative content analysis (Graneheim & Lundman, 2004) was used in the analysis.

### Participants

The participants comprised 18 children aged 7–9 from different areas of Sweden (eight girls and ten boys). They were all children of alcoholics, some of whom also were substance abusers, and all had one parent who had participated in treatment for their addiction at a treatment center. Most of the children lived with both their parents, but some of them had an alcoholic parent who lived elsewhere. All children were affected by the fact that they are COA.

The staff of the treatment center approached the parents who were undergoing treatment for addiction at the treatment center and asked if they had a child aged 7–12. If the answer was yes, they offered the parent the option of having their child or children participate in the Children’s Program, which was adapted in Sweden from the Betty Ford Children’s Program in the United States (Moe, Johnson, & Wade, 2008). The program is a 4-day educational program for children aged 7–12 who have a parent with an addiction problem.

At the same time as being informed about the Children’s Program, the staff also informed the parent about the research. The project managers of the Children’s Program gave more information about the program and about the research, and received permission from the parent for the researchers to send more detailed information about the research project along with letters of consent. These letters were designed in a suitable language for the children and for each of the parents. Informed consent was obtained from both parents of the child in writing, and from the child in writing and verbally. The Ethics Committee in Uppsala (ref: 2015/193) approved the study.

### Procedure

Interviews with the children aged 7–9 were conducted when the families arrived to participate in the Children’s Program. The children, who had all signed the letter of consent, were asked if they were willing to talk with one of the researchers for a short while in a private room before the program started. The children were informed verbally as well as in the information letter that nobody except the researchers would know about their answers, and that they could end the conversation and refuse to answer questions without any consequences. They were invited to join the program regardless of their choice over participating in the research. All children agreed to participate. One child did not participate due to parents’ reluctance. All children received two cinema tickets. Three researchers interviewed the children individually. These researchers were all used to talking to small children. One of the researchers, who performed most of the interviews, worked as a school nurse for several years, and had the responsibility for all researchers to keep a high ethical standard when interviewing the children.

The interviewer began by reading a vignette to the child about a girl, ‘Anna’, or a boy, ‘Peter’ (depending if the child being interviewed was a girl or a boy). The vignette was very short, and only told the child that ‘Anna’/‘Peter’ brought a friend home with them, but when they arrived, they heard their parents having a fight and seeming to be drunk (see Moe et al., 2007). Vignettes can be suitable to use when asking children to talk about sensitive topics (Barter & Renold, 2000; Hill, 2015). The children could talk about ‘Anna’ or ‘Peter’ in the vignette, without being specifically asked about their own situation. Some of the children stuck to the story, but others referred to their own situation. Following the vignette, the children were asked questions: “How do you think that ‘Anna’/‘Peter’ felt?”, “What do you think about ‘Anna’s’ or ‘Peter’s’ feelings inside her/his body?”, “According to you, what did ‘Anna’/‘Peter’ do?” and “What might a good life as an adult be?” Probing questions followed, such as “Please describe more”, “What do you think happened then?”

The children were also asked to describe their alcohol-abusing parents. The fact that the vignette implied that the researcher knew that fighting could happen in a family, especially when a parent had been drinking alcohol, could have made the children feel more open to tell their story. The interviews each lasted between 10 and 25 min, and were audio-taped with the permission of the children. The children were assigned fictitious names by which they are referred to in this article.

### Data Analysis

Analysis of the 18 interviews followed the procedure described by Graneheim and Lundman (2004). This method includes several steps. First, the unit of analysis is chosen; here, this consisted of the whole interview with each child. Second, meaning units are chosen, describing one meaning from the child at a time, and these are then condensed or shortened. Third, codes are used to abstract and label each condensed meaning unit. Fourth, the codes are compared in terms of their similarities and differences, and divided into subcategories, which are then clustered into categories. One or more themes are formulated from the underlying meaning.

The interviews were transcribed verbatim and read. One interview at a time was analyzed by choosing meaning units which were then condensed and coded. The codes were reviewed for similarities and differences, and assigned to subcategories. The subcategories were also compared in terms of their similarities and differences, and assigned to categories which were compared in the same way. The subcategories made the category concrete. Two members of the research team (AT and KD) analyzed inductively the first 11 interviews in this way, each of whom analyzed all of the 11 interviews. After that, the two researchers compared the subcategories and categories based on similarities and differences from the two separate analyses. They discussed the subcategories and categories, and made some adjustments before consensus was reached. For example were the names of the categories thoroughly discussed. The rest of the interviews were analyzed deductively in accordance with the categories previously established; the two researchers found no additional categories and made no changes. Finally, a theme was created. This was done when the researchers discussed all the interviews, searching for the meaning of the material as a whole (Table 1). The whole research team discussed the subcategories, categories and theme, and consensus was reached without any changes.

**Table 1:** Theme, categories, and subcategories showing the consequences of parental alcohol abuse for children of alcoholics from the point of view of 7–9-year-olds

Theme	Categories	Subcategories
Sadness and hope for change in a loving family	Feeling sad when my parents are fighting	Sensations in my body
		Showing sadness
		Not showing sadness
		Protecting my parents
	Trying to control the situation	Dissociating or removing myself from the fight
		Telling my parents to stop
		Seeking company
		Acting to prevent quarreling and fighting
		Suggesting ways to end the fight
	Having bad experiences	Fear that other people will know
		Parents drinking alcohol
		Being disappointed
	Wishing for change	No alcohol, no quarreling/fighting
		Having fun and a good life in the family
		Seeing my parent more often
	Despite problems, doing things together with loving parent	Having fun at home or away from home
		Everyday life is fine when things are going well
		My parent is nice

To ensure trustworthiness, the credibility, dependability, transferability and confirmability of the research can be discussed (Lincoln & Guba, 1985). By asking children with adequate experiences of the consequences for children with an alcoholic parent, we believe that credibility has been achieved. Dependability was established by using the same story in the vignette for all the children. The children in the current study told us about the consequences in their lives, and this might be possible to transfer to children in similar situations. Confirmability was established by using peer checking (Patton, 2015), by having the whole research group participate in the discussions of the analyses.

## Results

The results show the consequences for a child of having an alcoholic parent from the point of view of 7–9-year-old COA. When the children heard the vignette about the child who came home together with a friend, found their parents having a fight, and wondered if their parent was drunk, they recognized the situation. Most of the interviewees very quickly said “I felt…” or “When my parents…”, thus connecting the story with their own experiences. The children expressed different consequences of having an alcoholic parent, leading to five categories: “Feeling sad when my parents are fighting”, “Trying to control the situation”, “Having bad experiences”, “Wishing for change”, and “Despite problems, doing things together with a loving parent”. These five categories together formed a theme: “Feelings of sadness and hope for change in a loving family” (Table 1).

### Feeling Sad When My Parents are Fighting

Most of the children said that they thought the child in the vignette felt sad, when they came home and found their parents fighting. The questions thereafter comprised what they meant by “sad”; how did it feel and what else they thought of. The children’s answers are here divided into four subcategories: *sensations in my body, showing sadness, not showing sadness*, and *protecting my parents*.

According to the children, sadness could be felt in the body. It could be felt in the throat, like feeling strangled when trying not to cry, or in the stomach, like a bad feeling or stomach ache, or having a hole in one’s stomach. Another place where several of the children said they felt sadness was in the heart; their hearts felt empty when they were ashamed, or they felt that their hearts were breaking or hurting. Simon said that “sometimes it hurts inside.” And when the interviewer asked where it hurts, he answered “in my heart”.

Some of the children said that they clearly showed when they felt sad, which could be manifested as being angry or even “awfully angry”. One way was to slam their hands into things to make their parents understand that they were feeling bad. Crying was another way of showing sadness: “You get sad and you cry, and tears come out.” (Frederic).

However, not all children wanted to show how sad they felt, and some hid their feelings for other reasons. They expressed that they felt really bad, and that it also could be very embarrassing hearing their parents fighting, especially in front of friends. They might sometimes be ashamed of their parents. Even when they did not cry, it could be very difficult to think about good things. Paul said: “it’s almost like crying, you can’t think about nice things or anything like that”.

The sadness that the children felt could also be expressed by a feeling of protecting or caring for their parents, for example by not admitting how sad they felt, or by avoiding telling anybody how things were at home. Sandra said that she doesn’t “want anybody else to know. It’s like, it’s my family it’s all about. So I don’t want anybody else to know what’s happening in my family.”

The children were strongly affected by their parents’ quarrelling and fighting, and they used different strategies to deal with the sadness they felt.

### Trying to Control the Situation

The children had different ways of controlling the situation when they experienced quarrelling or fighting, here divided into five subcategories: *dissociating or removing myself from the fight, telling my parents to stop, seeking company, acting to prevent quarrelling or fighting*, and *suggesting ways to end the fight*.

Some of the children clearly dissociated and sometimes removed themselves from the situation by leaving the house, or by walking into their own room and shutting the door. They gave examples not only how to leave the room, actually removing themselves from the situation. They also gave examples how to dissociate oneself, by trying to think about something else, and trying not to hear or care what the fight was all about. One way was watching television to give themselves something else to think about. Another suggestion was trying to think about positive things, not bad things, and avoiding listening to the fight. Raymond suggested that you could “do something calm”, and he gave an example: “Just watch a movie or something like that.”

Other suggestions the children made for controlling the situation were to become very angry when their parents fought, which could influence their parents to stop the fight, or to show their parents that they felt sad. Donald suggested that one “can go out, or tell his parents […] You have to stop fighting!” The children felt that it was possible to tell their parents to stop quarrelling and fighting, though it might be important to wait to tell them this until they were alone and their friends could not hear.

Another way to handle the situation was to talk to somebody; that is, to seek company. Some of the children knew who they could call or go to if their parents started to fight—perhaps a grandmother or siblings. They also suggested talking to a teacher at school, or the school counselor. For some of the children, another possibility was to go to a friend, and maybe to talk with a friend’s mother. “He can go to his friend” (Jonathan).

The children described a couple of strategies to prevent their parents from drinking alcohol or using drugs in ways that led to quarrelling and fighting. Some of the children had thought about the problematic situation and how to make the situation better. One example was to hide their parent’s money or car keys so that they could not go to the liquor store. They could also act “grown-up” in other ways, for example when discussing the parent’s friends with the researcher.

Another strategy was to be “mean”, perhaps in an attempt to draw attention away from the quarrelling and fighting between the parents and from the problems within the family.

- Paul: When I was two or so. Then I learnt how to be mean, and then I was mean all the time.
- Interviewer: Were you mean all the time?
- P: Mmmm.
- I: How were you when you were mean?
- P: In school and at home.
- I: OK, why do you think you were mean?
- P. Because I learnt from my mom and dad.
- I: OK.
- P: Because they were fighting right in front of me.
- I: Yes, I understand. How were you when you were mean? What did you do?
- P: I just learnt more about how to be mean. I was mean every day. Then I learnt more and more and more.
- I: Did you think that you were mean, or did other people think you were mean? [child waves fists in air as if fighting] Oh, oh, oh, my god. What did you do? Were you fighting, or?
- P: Yes, I learnt more every time, and when someone was knocked down on the floor it made me so happy.
- I: OK, how are you now?
- P: Doesn’t feel so nice … to be mean.

The children suggested other solutions for how the fighting between the parents could stop; for example, if one of their parents left the house for a while, and came back later, or even stayed away for a longer time. In their experience, things also got better after their parents had separated from each other, and they thought it was a good thing for the alcoholic parent to start treatment.

- Karen: Well, then someone can do what my mom’s doing.
- Interviewer: OK, how do you mean, what your mom’s doing?
- K: To come here and stay here.
- I: To be in treatment.
- K: And then the other parent can stay with the children at home.

The children acted in different ways when trying to control the quarrelling and fighting between their parents, sometimes by preventing or interfering in the situation, and sometimes by physically removing themselves from the situation.

### Having Bad Experiences

The quarrelling and fighting between parents led to bad experiences, which are described here in three subcategories: the children experienced *fear that other people would know*, for example when bringing a friend home, they had seen their *parents drinking alcohol*, and they had a feeling of *being disappointed*.

The main reason why the children feared that other people would know was that they did not want other people to know about their parent’s alcoholism or drug addiction. A friend might tell their classmates that their parent had been drinking, which the children felt was not good at all. The children could feel embarrassed, or even be ashamed of one or both of their parents. Another possibility could be that their friend might ask why their parents were fighting. This could lead to rumors at school, which the children wanted to avoid.

- Interviewer: Would ‘Peter’ be sad because his friend told others? Why would he be sad about that, do you think?
- John: Mmm. He’d be ashamed.
- I: He’s ashamed?
- J: For having a father like that.
- I: How does it feel to be ashamed?
- J: No fun at all.
- I: No, of course not.
- J: And everybody at school knows that my father… Everybody talks about it.
- I: OK, how does it feel for you, that everybody talks about it?
- J: No fun at all.

The children had experienced the drinking at home, or they heard about it from the other parent. Sometimes the alcoholic parent was in a good mood and sometimes in a bad mood. One parent might start a fight with the other. The children were observant of the behavior of the abusive parent, which could be very different from when the parent was sober. Raymond described that “sometimes she drinks, but I notice it anyhow, even though I haven’t seen it […] …she acts a bit funny […] not the same as usual.” Although these children haven’t actually seen the parents drink at all times, they know when they do. It seems very difficult for parents to keep the drinking a secret. The children are very sensitive.

Sometimes the children felt disappointed with their parent, for example when the parent was supposed to spend time and do things together with the child, but preferred to talk or play on their cell phone.

- Karen: But now when she starts, she hasn’t got time for me, she just talks on her cell phone and so on.
- Interviewer: How does that feel to you?
- K: I get sad and angry. So I keep calling out to get her attention, because I wonder…
- I: When you do things together, how is it then?
- K: She brings her cell phone anyhow. And sends and sends…

The children all had bad experiences of the consequences of their parents’ alcohol consumption. They often kept it a secret, and they could experience disappointment towards their parents.

### Wishing for Change

The children had vague ideas about what they wanted in terms of change for the better and for them to have a good life as adults themselves. However, they did have some suggestions for what was important to them, here divided into three subcategories. *No alcohol, no quarrelling*/*fighting* was the most important thing, and the next most important was *having fun and a good life in the family*. The children who seldom saw their parent wished to *see their parent more often*.

According to the children, it would be much better if their parent did not drink alcohol; they should not do that, and they should not fight. The children also said that no adults should drink, including themselves when they got older, in order to have a good life as an adult. Sophia said: “Why is there beer really?” The children wished they would not hear of any more fighting, and hopefully be able to forget all about it. If the parent would forget about alcohol, everything would be fine.

- Interviewer: What will happen to that lump in your stomach?
- Cathy: It might loosen when you’re happy and not worried that it’ll happen again. Sometimes you can be very worried that it’ll happen again and again and that it’ll go on like this all the time. But then if you know that your parent’s getting help, and that they feel much better now, then the lump will loosen. So you’ll feel much better.

The children wished that their parents would be friendly towards each other, for the sake of their family; they were happy when their parents were friends. The children wished that they could all live together and have fun together. “You can be happy, play, ask your parents if they want to play. Nothing else.” (Jonathan).

The children who seldom saw their parent clearly expressed their sadness over this, and wished for more contact. They had hope for the future if their parent stopped drinking alcohol.

### Despite Problems, Doing Things Together with a Loving Parent

In spite of the bad consequences that these children experienced, they had happy memories of their alcohol or drug abusing parent. Their thoughts are divided here into three subcategories: *having fun at home or away from home, everyday life is fine when things are going well*, and the overall view that *my parent is nice*.

The children spoke about the fun things they often did with their parents. At these times, everyone was happy and in a good mood. At home, the children and parents played games in or out of the house. “We usually go out, playing on the swings or buying ice cream. Sometimes we’re indoors playing” (Sarah). The children wanted to be together with their parents, and that was what was important; it was less important exactly what they did, as long as they did it together. Some went swimming, and some went on road trips. The children expressed that they had a good time and were content being with their parents when they had not been drinking alcohol.

The children described everyday life as being all together in a cozy home. They had no extraordinary wishes, but could have a good time just doing ordinary things, “watch television, maybe eat something” (Stephen).

It was particularly the children who seldom saw their parent who expressed that the parent was very nice and kind. However, this overall view was expressed by all of the children when describing their parent. Camilla: said that “… she’s very kind […] She asks us if we want to go out shopping, but yes, she’s kind in many different ways.”

Despite the bad consequences of their parents’ drinking, and the fact that some of them seldom saw their parents, the children viewed their parents with love and affection. They wanted to spend time with their parents, and they thought they had a good time with their parents at home and elsewhere.

## Discussion

These 7–9-year-old children, all of whom had a parent who abused alcohol, have told about the consequences of having an alcoholic parent, from their point of view. They knew a great deal about their situation and were very aware of what this meant for the family. They could talk about their feelings, their strategies for handling the situation, and their hopes for the future. Our results show that these children were accustomed to the idea that their parents would fight when one of the parents had used alcohol. Some of the children cried and showed their feelings, but others hid their feelings and did not show them to their parents. Most of these COA had a feeling of sadness which they could feel in their body. Their feelings were expressed in different words, but consisted of almost the same emotions. Adolescent COA have described their experiences in previous research (Hill, 2015; Murray, 1998; Templeton et al., 2009). The adolescents showed that they had a lot of knowledge about their parent’s addiction, but they were very cautious of what to tell an outsider. Mostly they shared experiences of the parent’s treatment, which did not involve the rest of the family. These adolescents had experiences of moving around a lot (Hill, 2015). Other adolescents questioned their own worth, due to shame and guilt (Murray, 1998), and in a study by Templeton et al. (2009) adolescents told about their lives as being inconsistent and uncertain. The current study shows that COA in middle childhood, only 7–9 years old, can express feelings and describe the consequences for them having an alcoholic parent. Middle childhood is a period when children need to understand what is happening around them, and they have a need to understand themselves (Schofield, 2006). These children were well aware of the connection between the parent’s alcohol abuse and the child’s feelings of sadness, leading to feelings of disappointment towards parents. Therefore it is important to provide support to COA in middle childhood designed for their developmental stage.

The children in the current study showed that they wanted to control the situation when fighting occurred between their parents. Some of the children dissociated and sometimes removed themselves from the fight by leaving the room or the house. Trying to think about nice things or watching television to shut the fighting out was one way to take responsibility for themselves during the fight. It also seems that some of the children tried to protect their parents from understanding that they were hurting their children. The children had ideas about asking their parents to stop fighting, and planned to talk about the matter when they found their parent alone. This is an important aspect to pay attention to, as it might be a way that children act like adults and take too great a responsibility for the family. This is sometimes called parentification (Hedges, 2012; Vernig, 2011). Parentification can also be seen in the current study, when the children took responsibility by hiding their parent’s money or car keys to prevent them from buying alcohol. Wells and Jones (2000) discuss the relationship between shame and parentification, and note that COA who take on an adult’s role in the family have a higher risk of feeling ashamed of themselves as adults. It is possible that COA who do not take on this role are more resilient than those who take too much responsibility (Godsall, Jurkovic, Emshoff, Anderson, & Stanwyck, 2004). Murray (1998) reported on adolescent COA’s feelings of shame and guilt, which led to the adolescents questioning their own worth. When developing a conscience during middle childhood, feelings of shame and guilt may occur (Schofield, 2006). It may be important to give special support to COA in middle childhood (7–9 years old) to give them the opportunity to avoid feelings of shame and guilt because of their parent’s addiction. Earlier studies have indicated that witnessing parental violence has a strongly negative impact on children which continues into their later lives (Zhu & Dalal, 2010; WHO, 2002). The current study adds to this research, as it shows how the children planned to ask their parents to stop fighting. These 7–9-year-old COA tried to control the situation in their alcoholic families, and took on a responsibility that should have been taken by their parents.

It has been suggested that COA play different roles in the family. Adult COA have described how they acted as children (Black, 2001; Wegscheider-Cruse, 1989). These roles have been criticized for being too strict (Vernig, 2011). The present study indicates that the behavior typical of the “adjuster” (Black, 2001) or the “lost child” (Wegscheider-Cruse, 1989) is also expressed by 7–9-year-old children from their own point of view. They dissociated and sometimes removed themselves from the situation and tried to protect themselves. The roles of the “responsible child” (Black, 2001) or the “family hero” (Wegscheider-Cruse, 1989), also known as parentification, are supported in the current study. Some of the children in the current study also demonstrated the “acting-out child” (Black, 2001), when trying to draw the attention to themselves by “being mean”. Thus the current study on 7–9 years old COA, has confirmed the same sorts of COA behavior which adult COA have recalled that they have previously displayed (Black, 2001; Wegscheider-Cruse, 1989; Vernig, 2011). This is one of the important findings of the current study, indicating the need for early support to COA.

The results indicate how the COA wanted to have fun and a good family life. They wanted to play with their parents and friends. However, it should be remembered that the families in the current study cannot be generalized to every family with an alcohol or drug abusing parent. These parents had understood and taken responsibility for the consequences of their alcohol or drug abuse by accepting treatment at a treatment center. They had also understood that their children needed help and support to allow them to handle the situation in the best possible way, by joining a program specially designed for children. In this way, they had shown their children that they saw them as important and worthy of a good life. This might be one of the reasons why the children had such a positive view of their abusing parent. They had both suggestions and experiences of nice and fun things happening in the family. Parents and family are very important for COA, as for any other child or adolescent (Bronfenbrenner, 1979, 2000; Johansson et al., 2007; Tinnfält et al., 2015; United Nations, 1989). The children in the current study wanted to do ordinary things, mainly with their parents and family. Thus the current findings support the existing literature on how children find their parents and family very important. This indicates that supporting programs should address children and parents. The children wished for a future free of alcohol, both for themselves and for their parents, and for a future with no fighting. This is interesting in itself, as COA are at higher risk of becoming alcoholics themselves (Johnson & Leff, 1999). The children felt sadness over the current situation in their families, but still had hope for change.

A strength in this study is that COA as young as 7–9 years old have told about their experiences. To the authors’ knowledge there is not much known about as young COA describing their situation. Another strength is that as many as 18 7–9-year-old children have participated in the study. These children were not afraid of telling their stories, because they felt confident that their parent agreed to and wanted their participation in the study. This might have made the children reveal situations at home that they usually do not speak of. On the other hand it might be a limitation, as these parents obviously wanted the best for their child, as they themselves were undergoing treatment and wanted their child to join a special program. This is not the case for all COA. For some COA the rule of secrecy is very important, and they have not had the opportunity to join a program, as the children have in this study. More interviews, including children whose parents have not acknowledged their addiction, could have strengthen the study even more. Still, the results may be transferred to other COA, who live under similar circumstances.

### Implications

This study has some implications for the future. The results suggest that it is important to listen to children and their experiences, and that children are willing to tell when they are asked about their family situation. Children of middle childhood (7–9 years old) should be listened to, and taken seriously over their experiences and emotions related to being a COA. They understand a lot of what is happening in the family, and take responsibility for themselves and for their family. Some of them even take the role of an adult, so-called parentification. The results also suggest that more support should be provided to COA, as they know so much about their situation and they involve themselves by intervening in the situation of parents under the influence of alcohol. Support should be offered to children of middle childhood in a variety of ways, individual support, group support, family support. When parents take responsibility for their abuse by accepting treatment, it is of great importance that their children are supported. These children should be relieved of the pressure of taking responsibility for themselves and for their parents. There is a great need for interventions to support COA, both for adolescents and for younger children. In this study we have learnt how children as young as 7–9 years perceive or experience the situation. Further studies should address how the children perceive the support that they receive.

## Conclusion

COA as young as 7–9 years old have in this study told about their experiences of having an alcoholic parent after listening to a short vignette about parents being drunk and having a fight. They connected the story with their own experiences, and could tell about sadness but also hope for the future. They told how they took on a great responsibility for their parent’s addiction by trying to control the situation. The results suggest that it is important to listen to children and their experiences. The children have taken on a responsibility that should have been taken by their parents. Children should be relieved of the pressure of taking responsibility for themselves and for their parents, which demands for social support. Further studies should address how the children perceive the support that they receive.
